# Barriers and Predictors of HPV Vaccine Uptake Among Female Medical Students in Saudi Arabia: A Multi-Center Cross-Sectional Study

**DOI:** 10.3390/healthcare13192408

**Published:** 2025-09-24

**Authors:** Hanadi Bakhsh, Sarah Ali Altamimi, Falak Nasser Aldosari, Lujain Hatim Aljohani, Sarah Abdulrahman Alali, Nujud Ibrahim Almutlaq, Norah Khalid Alrusaini, Shuruq Munif Alshammari, Yara Abdulaziz Alsuhaibani, Shatha Abdulwahab Alshehri

**Affiliations:** 1 Department of Obstetrics and Gynecology, College of Medicine, Princess Nourah bint Abdulrahman University, Riyadh 11564, Saudi Arabia; 2 College of Medicine, Princess Nourah bint Abdulrahman University, Riyadh 11671, Saudi Arabia; saraltamimi@outlook.sa (S.A.A.); falaknn3@gmail.com (F.N.A.); lujain1492@gmail.com (L.H.A.); saraalal99@hotmail.com (S.A.A.); nujudapple@gmail.com (N.I.A.); nouraalresaini9@gmail.com (N.K.A.); shurooqmunif2000@gmail.com (S.M.A.); yara.aziz.273@gmail.com (Y.A.A.); 441002302@pnu.edu.sa (S.A.A.)

**Keywords:** HPV vaccination, cervical cancer prevention, medical students, vaccine hesitancy, Saudi Arabia, gynecological oncology, public health education

## Abstract

**Background/Objectives:** Low HPV vaccine uptake persists in Saudi Arabia despite improving awareness. This study aimed to assess the level of awareness, knowledge, and uptake of the human papillomavirus (HPV) vaccine among female medical students in Saudi Arabia, and to identify key demographic, academic, and informational factors that predict vaccination behavior. It also sought to explore perceived barriers influencing vaccine acceptance within this population. **Methods:** A multi-center cross-sectional survey recruited 246 female medical students from five Saudi universities using convenience sampling. An anonymous, structured, and validated questionnaire assessed HPV/vaccine knowledge, attitudes, uptake, and perceived barriers. Data were analyzed using descriptive statistics, *t*-tests, ANOVA, correlation, and logistic regression. **Results:** Overall, 82.9% of participants had heard of HPV and 78.9% knew of the vaccine, but only 10.3% demonstrated high vaccine-specific knowledge. While 69.5% expressed willingness to be vaccinated, only 22.8% had received at least one dose. The most reported barriers were perceived lack of necessity (45.3%), abstinence from sexual activity (41.3%), and safety concerns (34.7%). Logistic regression indicated that higher academic year and higher vaccine-specific knowledge significantly predicted vaccine uptake (*p* < 0.001). **Conclusions:** Despite high general awareness, low vaccine-specific knowledge and cultural framing constrain HPV vaccine uptake among future prescribers. Universities should integrate cancer-prevention framing into curricula, strengthen female-to-female counseling, and provide on-campus vaccination opportunities. Addressing knowledge gaps and sociocultural barriers is critical to improving HPV vaccine coverage in Saudi Arabia.

## 1. Introduction

Human papillomavirus (HPV) is one of the most prevalent sexually transmitted infections (STIs) globally and a major etiological factor in cervical cancer and other anogenital and oropharyngeal malignancies [1]. The World Health Organization (WHO) reports that virtually all cases of cervical cancer (99%) are attributable to persistent infection with high-risk HPV strains, particularly types 16 and 18 [2].

Vaccination against HPV represents a cornerstone of global cervical cancer prevention efforts. Since their introduction, the available vaccines—bivalent, quadrivalent, and nonavalent formulations—have demonstrated strong efficacy in preventing infection with oncogenic HPV types [3,4]. The Centers for Disease Control and Prevention (CDC) and WHO recommend vaccination for girls and boys starting as early as 9 years of age, ideally before the onset of sexual activity [5]. Despite strong global evidence supporting its safety and efficacy, uptake of the HPV vaccine remains uneven, especially in low- and middle-income countries, where awareness is limited and cultural and systemic barriers persist [6,7].

In Saudi Arabia, the burden of HPV-related disease is becoming increasingly apparent. Although cervical cancer ranks as the eighth most common cancer among Saudi women overall, it is the third most common gynecological malignancy and causes approximately 179 deaths annually [8]. Notably, the majority of these cases occur in women between the ages of 15 and 44, underscoring the need for early and effective prevention strategies [9]. The Kingdom introduced the HPV vaccine into its national immunization schedule in 2010, targeting females aged 11 to 26. However, vaccination coverage remains disappointingly low, with estimates suggesting uptake rates as low as 4% in some regions [10]. Cultural sensitivities, misconceptions regarding the vaccine’s purpose, and insufficient knowledge among the public and even healthcare professionals are major contributing factors [11,12].

Medical students, as future physicians and public health advocates, play a pivotal role in shaping societal perceptions and acceptance of vaccines. Their knowledge, attitudes, and behaviors regarding HPV vaccination are critical not only for their own health but also for their future capacity to recommend and administer the vaccine to patients [13]. Unfortunately, existing literature reveals gaps in vaccine literacy even among this highly educated group. In studies conducted in Saudi Arabia and other Middle Eastern countries, many medical students demonstrated limited understanding of the vaccine’s indications, dosing schedule, and safety profile [14,15]. Additionally, sociocultural factors, including stigmatization of sexually transmitted infections, further complicate vaccine acceptance [16].

A cross-sectional study in Jeddah found that only 42% of male medical students had heard of the HPV vaccine, and just 14% correctly identified its target age group [17]. Another study conducted among health sciences students at a Saudi university showed that while general awareness of HPV was relatively high, fewer than 20% of participants had adequate vaccine-specific knowledge [18]. These findings are concerning given the central role these students will play in vaccine promotion and cancer prevention. Furthermore, previous research suggests that gender influences attitudes and knowledge regarding HPV vaccination, with female students often demonstrating higher awareness but facing additional sociocultural barriers to access and uptake [19].

Despite the growing body of research on HPV vaccination in the Saudi population, there is a notable paucity of studies focusing specifically on female medical students—a group that is simultaneously at risk of HPV-related diseases and well-positioned to serve as future health educators and vaccine advocates. Their perspectives are particularly relevant in the Saudi context, where female healthcare providers often serve as the primary source of reproductive health information for women, due to gender norms that may limit open communication between female patients and male providers [20].

In Saudi Arabia, modesty norms and religiously informed values around sexuality can shape perceptions of STI-related prevention, sometimes leading to abstinence-based reasoning (‘no perceived need’) and concerns about moral signaling that suppress vaccine-seeking despite awareness. Framing HPV vaccination primarily as cancer prevention and ensuring private, female-to-female counseling pathways can reduce stigma and increase acceptability within this context. Engaging trusted educators and community figures may further normalize vaccination without conflicting with cultural expectations.

Understanding the knowledge levels, attitudes, and vaccine behaviors of female medical students is essential for informing educational interventions, shaping health communication strategies, and ultimately improving vaccine coverage. In particular, identifying the barriers that impede HPV vaccine uptake—such as perceived lack of necessity, absence of sexual activity, safety concerns, religious beliefs, and lack of physician recommendation—is vital for tailoring interventions that are culturally appropriate and contextually relevant [21].

Targeting female medical students is strategic for two reasons: first, they are at the age window where HPV vaccination yields maximum cancer-prevention benefit; second, as future prescribers and counselors, their literacy and personal uptake shape clinical recommendation norms, peer influence, and public acceptance—a multiplier effect that extends beyond this cohort. Generating baseline data in this cadre enables rapid, curriculum-embedded interventions (e.g., counseling skills, on-site vaccination) that can translate into improved population coverage. Community-based probability samples remain essential and are a planned next step to complement these profession-focused findings

**Aim of the Study**

The aim of this study is to assess the level of awareness, knowledge, and uptake of the human papillomavirus (HPV) vaccine among female medical students in Saudi Arabia, and to identify key demographic, academic, and informational factors that predict vaccination behavior. Additionally, the study seeks to explore the perceived barriers influencing vaccine acceptance within this population.

**Research Questions**

1. What is the level of awareness and knowledge regarding HPV infection and its vaccine among female medical students in Saudi Arabia?
2. What is the rate of HPV vaccine uptake among this population?
3. What are the most commonly reported barriers to HPV vaccine acceptance among female medical students?
4. Which demographic, academic, or knowledge-related factors significantly predict HPV vaccine uptake?

## 2. Materials and Methods

### 2.1. Study Design

A multi-center descriptive cross-sectional design was employed to comprehensively investigate awareness, knowledge, and uptake of the Human Papillomavirus (HPV) vaccine among female medical students in Saudi Arabia. The choice of a cross-sectional approach allowed for effective capture of existing behaviors, knowledge gaps, and perceptions regarding HPV vaccination at a defined point in time, providing valuable baseline data for policy and educational interventions. A multi-center convenience approach enabled timely recruitment across five geographically dispersed colleges. We acknowledge potential selection and non-response biases (e.g., over-representation of senior or higher-GPA students) and therefore interpret estimates as baseline and hypothesis-generating

### 2.2. Study Setting

This study was conducted across five major medical universities strategically located to represent diverse geographical regions within Saudi Arabia, ensuring comprehensive national coverage. The selected institutions included Princess Nourah Bint Abdulrahman University (PNU) and King Saud University in Riyadh, King Abdulaziz University in Jeddah, Imam Abdulrahman Bin Faisal University in the Eastern Province, and Taibah University in Al-Madinah Al-Munawwarah. These universities are recognized for their substantial contributions to medical education and serve as influential centers in shaping future healthcare professionals.

### 2.3. Sample and Sampling Technique

The study targeted female undergraduate medical students aged 21 years and older, enrolled in academic years one through five at the selected institutions. Inclusion criteria specified female gender, active enrollment in medical school, and voluntary informed consent for participation. Students from other health-related disciplines, those below 21 years, or individuals unwilling to consent were excluded. 

A convenience sampling approach was utilized due to logistical constraints and the extensive geographic dispersion of the participating universities. Sample size estimation was calculated using the Raosoft calculator, employing a 95% confidence interval and 5% margin of error with a presumed 50% response distribution. Based on this calculation, the minimum required sample size was 221 participants. To account for potential non-responses or incomplete data, the sample was increased, ultimately enrolling 246 eligible female medical students.

### 2.4. Data Collection Tools

Data were collected through a structured, validated, self-administered online questionnaire developed using Google Forms. The questionnaire was adapted from previously validated instruments utilized in HPV-related research conducted by Ahmad et al. (2023) [22], Algaadi (2024) [23], and Aldawood et al. (2024) [24], and modified to suit the specific objectives and cultural context of the current study. The questionnaire aimed to gather demographic details, assess knowledge regarding HPV infection and vaccination, evaluate vaccine acceptability and attitudes, and identify barriers influencing vaccination decisions. It consisted of four distinct sections:

1. **Demographic and Academic Information**:

This section aimed to obtain data on age, nationality, marital status, university affiliation, year of study, academic performance (GPA), smoking status, and vaccination history. These variables facilitated subgroup analysis and identification of predictors associated with HPV knowledge and vaccination uptake.

2. **HPV Infection Knowledge Assessment Tool**:

This tool assessed students’ knowledge regarding HPV infection, its transmission, associated health outcomes, and preventive measures. Adapted from previous HPV awareness studies, it comprised 17 multiple-choice items [25]. Each correct answer was awarded 1 point, while incorrect or “I don’t know” responses received 0 points, yielding a maximum possible score of 16 points. Knowledge was classified as low (≤7 points), moderate (8–12 points), or high (≥13 points). The tool exhibited strong internal consistency in pilot testing (Cronbach’s alpha = 0.81), indicating reliability. It underwent forward and backward translation into Arabic by bilingual public health experts to ensure linguistic and cultural validity, with subsequent validation confirming clarity and appropriateness for the Saudi context.

3. **HPV Vaccine Knowledge Assessment Tool**:

This tool, adapted to measure detailed knowledge of the HPV vaccine, including vaccine types, recommended administration ages, dosing schedules, safety profiles, and protective benefits [26]. Consisting of 12 items, scoring mirrored the infection knowledge tool, with correct responses assigned 1 point and incorrect or uncertain responses 0 points, yielding a maximum score of 11. Knowledge levels were categorized into low (≤5 points), moderate (6–8 points), and high (≥9 points). This tool demonstrated excellent internal consistency (Cronbach’s alpha = 0.84). Translation and validation procedures were identical to those employed for the infection knowledge tool, ensuring suitability for the targeted Arabic-speaking population.

4. **HPV Vaccine Acceptability and Attitudes Tool**:

Designed to evaluate vaccine acceptance, barriers, and behavioral intentions, this tool included five items adapted from the validated scale by Algaadi (2024) [23]. Items assessed willingness to receive the HPV vaccine, perceived barriers (e.g., safety, necessity, cultural issues), and intentions to advocate for vaccination among future patients [27]. Respondents could select multiple responses regarding barriers, facilitating a comprehensive understanding of attitudinal and perceptual influences.

All questionnaire components underwent rigorous review by a panel of three public health and epidemiology experts, ensuring content validity, relevance, and appropriateness within the Saudi medical education context.

To situate our findings internationally, we compiled a descriptive comparison of awareness, uptake, and barrier profiles among female university/health students in diverse settings (Supplementary Table S1).

### 2.5. Data Collection Procedure

The electronic questionnaire was disseminated via institutional mailing lists, student forums, and social media platforms (WhatsApp, Telegram) associated with each participating university. A cover letter explaining the study objectives, voluntary participation, confidentiality assurance, and instructions was provided at the outset. Data collection spanned a four-week period, during which biweekly reminders were issued to enhance response rates. Participation was voluntary, and informed consent was electronically recorded before questionnaire access was granted. The online questionnaire did not collect names, student IDs, contact details, or IP addresses. Participation was anonymous and voluntary, and only de-identified data were analyzed. Site recruitment and liaison were coordinated by trained female staff/student representatives to enhance cultural appropriateness.

### 2.6. Data Management and Statistical Analysis

Data were extracted from Google Forms into Microsoft Excel, cleaned for consistency and completeness, and subsequently imported into Statistical Package for the Social Sciences (SPSS) software, version 23.0. Descriptive analyses included frequencies, percentages, means, and standard deviations. Inferential statistics employed independent *t*-tests, one-way ANOVA with post hoc (Tukey) tests, Pearson correlation analysis, and logistic regression models. Statistical significance was predetermined at *p* < 0.05, facilitating precise identification of significant predictors of vaccine uptake. To mitigate collinearity, we retained academic year as the primary proxy for training exposure and omitted age from the final multivariable model. Pairwise checks indicated no high collinearity among retained predictors; inference was unchanged.

### 2.7. Ethical Considerations

Ethical approval was obtained from the Institutional Review Board (IRB) at Princess Nourah Bint Abdulrahman University, Riyadh, Saudi Arabia (IRB log number: 24-0816), prior to study initiation. Participants received comprehensive electronic consent forms detailing study objectives, voluntary nature of participation, privacy measures, anonymity of responses, and their right to withdraw at any stage without repercussions. Confidentiality of data was rigorously maintained, with access limited strictly to the research team. Data were used exclusively for academic and research purposes. No incentives were provided to participants, ensuring voluntary and unbiased participation

## 3. Results

### Participant Characteristics

A total of 246 female medical students from five Saudi universities participated in the study. The mean age of participants was 22.23 ± 1.69 years, ranging from 21 to 32 years. Most participants (98.4%) were Saudi nationals, predominantly single (95.9%), and non-smokers (88.2%). Participants were mostly senior students, with the largest group in their fifth academic year (37.4%). Academic performance was high, with the majority (61.0%) reporting excellent cumulative GPA scores. These demographic and academic details are comprehensively illustrated in Table 1.

Of the 246 participants, 82.9% reported prior awareness of HPV. Among those aware, detailed knowledge assessment yielded a mean score of 11.31 ± 2.86 out of 16 points. Most students (48.0%) demonstrated moderate HPV infection knowledge, while 41.2% exhibited high knowledge. Nonetheless, approximately one-tenth (10.8%) exhibited low levels of detailed HPV knowledge. These findings highlight the presence of substantial yet uneven knowledge distribution (Table 2).

Among students aware of HPV vaccination (78.9%), detailed vaccine-specific knowledge was notably lower (mean score: 5.51 ± 1.77 out of 11 points). Nearly half (47.4%) fell into the low knowledge category, with only 10.3% exhibiting high vaccine-specific knowledge. This indicates critical gaps in detailed knowledge despite widespread general awareness (Table 3).

The majority of participants (85.8%) identified medical education as their primary source of HPV information, followed by media sources (35.8%) and the internet (31.4%). Family and friends were the least reported source (17.6%), highlighting the prominence of academic institutions in disseminating health-related information among medical students (Table 4).

Although 69.5% of respondents expressed interest in receiving the HPV vaccine, actual vaccination uptake was substantially lower (22.8%). The main barriers identified included perceptions of the vaccine being unnecessary (45.3%), lack of sexual activity (41.3%), and safety concerns (34.7%). These attitudinal barriers underscore the urgent need for targeted educational interventions to address misconceptions and boost vaccination rates (Table 5).

Logistic regression analysis revealed that educational level (particularly fourth- and fifth-year students) and high vaccine-specific knowledge scores significantly increased odds of HPV vaccination. Fifth-year students showed approximately six times higher odds of vaccination compared to first-year students (OR = 5.92; *p* < 0.001). Moreover, each unit increase in vaccine knowledge scores increased the odds of vaccination by 1.36-fold (*p* < 0.001), emphasizing knowledge as a critical factor (Table 6).

**Figure 1:**
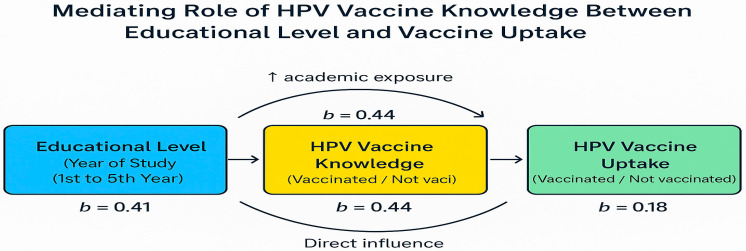
Mediating Role of HPV Vaccine Knowledge Between Educational Level and Vaccine Uptake.

The diagram (Figure 1) visually represents the hypothesized and statistically supported relationships between key variables in the study. It illustrates that educational level (measured by year of study) exerts both a direct influence on HPV vaccine uptake (b = 0.18) and an indirect influence mediated through HPV vaccine knowledge. The strength of the relationship between educational level and knowledge is notable (b = 0.44), indicating that higher academic progression is associated with improved understanding of HPV vaccination. In turn, vaccine knowledge strongly predicts vaccine uptake (b = 0.44), suggesting that informed students are more likely to accept vaccination. The presence of both direct and indirect pathways implies partial mediation, with knowledge acting as a critical mechanism through which education enhances health behavior. The diagram effectively clarifies the conceptual model tested in the study and reinforces the practical importance of early and targeted HPV education in medical curricula to drive behavior change.

## 4. Discussion

The present study provides important insights into the awareness, knowledge, and uptake of the HPV vaccine among female medical students across Saudi Arabia, identifying critical gaps between knowledge and actual preventive health behaviors. Although general awareness regarding HPV infection and vaccination was high among the study participants, detailed knowledge was notably deficient, reflected by moderate to low vaccine-specific knowledge scores. This discrepancy aligns with previous findings from international research [28,29] and highlights a prevalent educational gap that may hinder vaccine advocacy among future healthcare providers.

Our findings demonstrated that while over 82% of students were aware of HPV infection, fewer possessed comprehensive, vaccine-specific knowledge (10.3%). Similar trends were identified in other recent studies conducted in Saudi Arabia and neighboring Gulf countries, indicating that general awareness does not necessarily translate into detailed understanding of vaccine indications, schedules, and benefits [30,31]. Such knowledge deficits have critical implications for public health, as they undermine medical students’ ability to effectively communicate vaccine benefits to patients, potentially perpetuating misconceptions and vaccine hesitancy in broader communities [32].

Vaccine uptake among our participants was suboptimal, with only approximately 22.8% vaccinated, despite nearly 70% expressing a willingness to receive vaccination. This gap between intention and behavior is consistent with prior research, suggesting that perceived barriers significantly deter vaccination even among informed populations [33]. The primary barriers cited by students—perceived lack of necessity due to absence of sexual activity, safety concerns, and insufficient knowledge—are recurrent themes identified in previous international studies investigating vaccine hesitancy [34]. These persistent barriers highlight the urgent need for tailored, culturally sensitive interventions addressing local socio-cultural norms and religious sensitivities surrounding sexual health education [35,36].

Importantly, the logistic regression analysis in this study identified educational level and vaccine-specific knowledge as significant predictors of HPV vaccine uptake [37]. Students in advanced academic years (particularly fourth- and fifth-year medical students) exhibited substantially higher odds of being vaccinated, underscoring the importance of academic exposure in enhancing vaccine literacy and health-promoting behaviors [38,39]. This finding corroborates earlier research indicating that longitudinal exposure to medical curricula emphasizing preventive medicine and vaccine education increases acceptance and uptake of vaccinations [40]. As medical education progresses, students gain more detailed knowledge and experience, becoming more equipped to appreciate the clinical benefits and safety of HPV vaccination [41].

The mediating role of vaccine-specific knowledge demonstrated in our mediational analysis further emphasizes the strategic importance of educational interventions. This mediation indicates that educational progression positively influences vaccine uptake primarily through its impact on knowledge enhancement. Such results align with theoretical frameworks like the Health Belief Model and Social Cognitive Theory, emphasizing knowledge and perceived benefits as critical predictors of health behavior [42]. Thus, integrating detailed HPV education early within medical curricula could significantly address knowledge deficits and misconceptions, thereby promoting more proactive preventive behaviors among future healthcare providers [43].

Medical education emerged as the primary source of HPV-related information for most participants. However, considerable reliance on informal sources such as media and internet platforms was also evident. These sources, while accessible, pose significant risks due to misinformation and inconsistent messaging, a finding widely reported in other public health studies [44]. Therefore, academic institutions bear the critical responsibility of delivering structured, evidence-based information, counteracting misinformation, and fostering accurate vaccine-related knowledge and perceptions [1,45,46,47,48].

In Saudi Arabia, modesty norms and religiously informed values around sexuality can influence perceptions of STI-related prevention, potentially constraining disclosure and vaccine-seeking even when benefits are understood [46]. Such norms may promote abstinence-based reasoning (‘no need’) and moral signaling concerns that deter uptake. Framing HPV vaccination primarily as cancer prevention, ensuring female-to-female counseling, and engaging trusted community/religious figures may reduce stigma and normalize vaccination.

This study’s findings have several practical and policy implications. Primarily, enhancing vaccine-specific education within medical curricula should be prioritized to reduce HPV-related morbidity and mortality effectively. Furthermore, given identified barriers such as safety concerns and cultural perceptions related to sexual health, public health campaigns must adopt culturally sensitive and respectful communication strategies addressing local norms and values [47,49]. Engaging respected community and religious leaders in promoting vaccine acceptance could also facilitate the normalization and acceptance of HPV vaccination across different societal sectors [50,51].

### 4.1. Implications for Practice/Policy

Focusing on female medical students offers a high-leverage path to near-term impact because they are both at the age of vaccine benefit and on the cusp of becoming prescribers whose recommendations shape patient decisions. Medical schools can translate high willingness into actual uptake by embedding concise, competency-based teaching on HPV vaccine counseling within existing curricula, explicitly framing the vaccine as cancer prevention and rehearsing female-to-female counseling scenarios to address safety myths and modesty norms. Parallel structural supports should make vaccination easy and discreet: student health services can provide on-campus, privacy-assured clinics staffed by female vaccinators, with simple reminder–recall and same-day access after teaching sessions. Clear communication about eligibility, coverage, and out-of-pocket costs—presented in student portals and course sites—reduces uncertainty that often masquerades as attitudinal hesitancy. Faculty development is equally important so preceptors model proactive, non-judgmental recommendations during clinical rotations. Finally, culturally responsive outreach—co-developed with respected religious/community figures and female faculty champions—can reduce stigma and normalize preventive care, while routine monitoring of uptake and missed opportunities provides feedback for continuous improvement.

### 4.2. Future Research

To move beyond cross-sectional associations, a sequential explanatory mixed-methods program is warranted. Longitudinal follow-up of current cohorts can track transitions from intention to initiation/completion and quantify the durability of educational effects, after which focus groups or interviews can unpack the moral signaling concerns, abstinence-based reasoning, and safety narratives that surveys cannot illuminate. Cluster-randomized evaluations of bundled interventions—curricular training coupled with on-site vaccination—would test causal effects on uptake across colleges and allow head-to-head comparisons of messaging frames (cancer-prevention vs. STI-prevention). Broadening the sampling frame to include male medical students, non-medical faculties, and community participants will widen inference and reveal peer effects. Future instruments should measure structural access—insurance/coverage, eligibility awareness, clinic proximity, time and travel costs—to disentangle informational from logistical barriers, and report implementation outcomes (acceptability, feasibility, fidelity, and cost) to guide scale-up where effective.

### 4.3. Limitations

This multi-center study relied on convenience sampling and self-report within a cross-sectional design. As a result, selection and non-response biases may have favored more engaged or senior students, potentially inflating awareness and stated willingness, while social-desirability pressures in a culturally conservative context could simultaneously suppress perceived need and overstate favorable attitudes. The exclusive focus on female medical students constrains generalizability to male peers and the wider public. Moreover, the absence of socioeconomic and access measures—such as insurance coverage, eligibility awareness, proximity to services, and out-of-pocket costs—limits our ability to separate informational from structural determinants of uptake and may bias estimated associations in an uncertain direction. Finally, the cross-sectional design precludes any inference about temporality or causality.

## 5. Conclusions

Despite high general awareness, HPV vaccine–specific knowledge and actual uptake among female medical students remain low, with abstinence-based reasoning, perceived lack of necessity, and safety concerns reflecting powerful cultural framing. Because these students are future prescribers and health advocates, targeted educational and structural strategies within medical schools are both timely and impactful. Universities should integrate cancer-prevention framing into curricula, provide female-to-female counseling skills training, and offer on-campus vaccination with streamlined access pathways. Partnerships with trusted community and religious figures can further destigmatize vaccination and normalize preventive care. While our convenience, cross-sectional sample limits generalizability and causal inference, the findings identify actionable levers for improving uptake now and outline priorities for mixed-methods, longitudinal, and more representative studies—including male students and socioeconomic/access measures—to guide culturally attuned HPV vaccination initiatives in Saudi Arabia.

## Figures and Tables

**Table 1:** Socio-Demographic and Academic Characteristics of Study Participants (N = 246).

Characteristic	Categories	Frequency (n)	Percentage (%)
Age (Mean ± SD, years)	22.23 ± 1.69		
Nationality	Saudi	242	98.4
	Non-Saudi	4	1.6
Marital Status	Single	236	95.9
	Married	9	3.7
	Divorced	1	0.4
Academic Year	First-year	36	14.6
	Second-year	33	13.4
	Third-year	40	16.3
	Fourth-year	45	18.3
	Fifth-year	92	37.4
GPA	Excellent (4.50–5.00)	150	61.0
	Very Good (3.75–4.49)	82	33.3
	Good (2.75–3.49)	12	4.9
	Pass (2.00–2.74)	2	0.8
Smoking Status	Never	217	88.2
	Current	20	8.1
	Former	9	3.7

**Table 2:** Levels of HPV Infection Knowledge (n = 204).

Knowledge Level	Score Range	Frequency (n)	Percentage (%)
High	≥13	84	41.2
Moderate	8–12	98	48.0
Low	≤7	22	10.8

**Table 3:** Detailed Knowledge Levels about HPV Vaccine (n = 194).

Knowledge Level	Score Range	Frequency (n)	Percentage (%)
High	≥9	20	10.3
Moderate	6–8	82	42.3
Low	≤5	92	47.4

**Table 4:** Sources of HPV Information Reported by Participants (Multiple responses allowed, n = 204).

Information Source	Frequency (n)	Percentage (%)
Medical Education (courses)	175	85.8
Media (TV, Social Media)	73	35.8
Internet (websites)	64	31.4
Healthcare Providers	43	21.1
Family/Friends	36	17.6

**Table 5:** HPV Vaccine Acceptance, Uptake, and Perceived Barriers (n = 246).

Variable	Frequency (n)	Percentage (%)
Previously Vaccinated	56	22.8
Interested in Vaccination	171	69.5
**Barriers:** *(Multiple answers allowed)*		
Perceived unnecessary	34	45.3
Not sexually active	31	41.3
Safety concerns	26	34.7
Insufficient information	21	28.0
Lack of doctor recommendation	15	20.0

**Table 6:** Logistic Regression Predictors of HPV Vaccine Uptake (N = 246).

Variables	Odds Ratio (OR)	95% CI	*p*-Value
Age (years)	1.12	0.95–1.32	0.162
Second-year	1.74	0.52–5.85	0.370
Third-year	3.21	1.05–9.79	0.042 *
Fourth-year	4.28	1.47–12.46	0.008 *
Fifth-year	5.92	2.15–16.30	<0.001 *
HPV Vaccine Knowledge Score	1.36	1.18–1.57	<0.001 *

* Statistically significant at *p* < 0.05.

## Data Availability

The data presented in this study are available on request from the corresponding author. The data are not publicly available due to privacy and ethical restrictions.

## Supplementary Materials

The following supporting information can be downloaded at: https://www.mdpi.com/article/10.3390/healthcare13192408/s1, Table S1. HPV awareness, HPV vaccine awareness, uptake, and predominant barriers among university/health students across selected Islamic and non-Islamic settings. Refs. [28,52,53,54,55] are cited in the Supplementary Materials.
