# Supplementary material for: Barriers and Predictors of HPV Vaccine Uptake Among Female Medical Students in Saudi Arabia: A Multi-Center Cross-Sectional Study
Source: Healthcare (Basel). 2025 Sep 24;13(19):2408. doi: 10.3390/healthcare13192408 (PMC12523730; doi:10.3390/healthcare13192408)
Supplement: Supplementary file 1 [file healthcare-13-02408-s001.zip › healthcare-3815468-supplementary.pdf]

Supplementary Table S1. HPV awareness, HPV vaccine awareness, uptake, and predominant barriers among university/health students across selected Islamic and non-Islamic settings

Notes: NR = not reported; percentages are as reported in each study. Uptake refers to ≥1 dose unless otherwise specified. Where studies sampled mixed-gender students, overall rates are shown (female-specific figures were not consistently available). *In Cheema 2024, 77.7% had some-to-good knowledge about HPV/vaccination. †See reference list below for full citations.

Country/Setting	Population	N	HPV awareness (%)	HPV vaccine awareness (%)	HPV vaccine uptake (≥1 dose, %)	Predominant barriers (harmonized categories)	Source
**Saudi Arabia (current study)**	Female medical students	246	82.9	78.9	22.8	Perceived lack of necessity (45.3%); abstinence from sexual activity (41.3%); safety concerns (34.7%)	Current study
**Saudi Arabia (Riyadh)**	College students (mixed)	442	54.1	36.2	10.0	Lack of education/awareness reported as primary barrier (80.1%)	Alghalyini 2024†
**Qatar (Doha)**	University students (63% female)	398	77.7*	NR	6.3	Knowledge gaps; vaccination willingness contingent on provider recommendation (71% if recommended)	Cheema 2024†
**China (multi-city)**	Female university students	1438	NR	NR	23.6 (10.6% appointment; 84.4% willing)	Lower knowledge, trust, and risk perception associated with hesitancy	Chen 2024†
**Nigeria (Lagos)**	Students in a tertiary institution (mixed)	240	NR	15.4 (heard of HPV vaccine)	2.1	Unaware of vaccine availability/need (88.8% of unvaccinated)	Akande 2024†
**Brazil (Brasília)**	Medical student	379	NR	NR	21.1	Lack of advice/recommen	da Silva Wander

	s (mixed)					dation; male sex associated with lower uptake	ley 2019†

References for Supplementary Table S1:

- Alghalyini B, Zaidi ARZ, et al. Awareness and knowledge of human papillomavirus, vaccine acceptability and cervical cancer among college students in Saudi Arabia (2024).

- Cheema S, Abraham A, et al. HPV infection and vaccination: a cross-sectional study of knowledge, perception, and attitude to vaccine uptake among university students in Qatar (2024).

- Chen X, Wang L, Huang Y, et al. Risk perception and trust in the relationship between knowledge and HPV vaccine hesitancy among female university students in China (2024).

- Akande OW, et al. Human Papillomavirus vaccination amongst students in a tertiary institution in the Lagos metropolis (2024).

- da Silva Wanderley M, et al. Students' HPV vaccination rates… in medical school in Brasília, Brazil (2019).

- Current study (Saudi Arabia, female medical students).
